# 3-(3,4-Dimeth­oxy­benz­yl)chroman-4-one

**DOI:** 10.1107/S1600536813000925

**Published:** 2013-01-16

**Authors:** S. Shalini, C. R. Girija, Lalitha Simon, K. K. Srinivasan, T. V. Venkatesha, M. M. Jotani

**Affiliations:** a Chemistry Research Centre (affiliated to Kuvempu University), SSMRV Degree College, 4th T Block, Jayanagar, Bangalore 560 041, India; b Department of Chemistry, KMC International Center, Manipal University, Manipal 576 104, India; c Department of Pharmaceutical Chemistry, Manipal College of Pharmaceutical Sciences, Manipal University, Manipal 576 104, India; d Department of Chemistry, Jnana Sahyadri, Kuvempu University, Shankargatta 577 451, India; e Department of Physics, Bhavan’s Sheth R. A. College of Science, Khanpur, Ahmedabad, Gujarat 380 001, India

## Abstract

In the title compound, C_18_H_18_O_4_, the six-membered chroman-4-one ring adopts an envelope conformation with the C atom bonded to the bridging CH_2_ atom as the flap. The dihedral angle between the mean plane of the fused pyranone ring and the dimeth­oxy-substituted benzene ring is 89.72 (2)°. In the crystal, adjacent molecules are linked *via* C—H⋯π inter­actions.

## Related literature

For the biological activity and pharmaceutical properties of chromenes(benzopyrans) and a similar structure, see: Jasinski *et al.* (2010 ▶). For bond-length data see: Allen *et al.* (1987 ▶). For ring conformations, see: Cremer & Pople (1975 ▶)
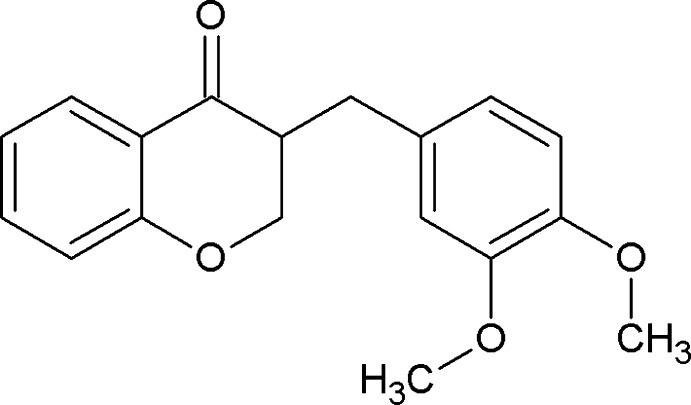


## Experimental

### 

#### Crystal data

C_18_H_18_O_4_
*M* *_r_* = 298.33
Monoclinic, 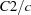

*a* = 30.414 (4) Å
*b* = 5.453 (3) Å
*c* = 20.661 (5) Å
β = 118.568 (3)°
*V* = 3009.4 (19) Å^3^
*Z* = 8
Mo *K*α radiation
μ = 0.09 mm^−1^
*T* = 295 K
0.30 × 0.20 × 0.20 mm

#### Data collection

Bruker Kappa APEXII CCD diffractometer
Absorption correction: multi-scan (*SADABS*; Sheldrick, 1996 ▶) *T*_min_ = 0.954, *T*_max_ = 0.991
13379 measured reflections
2802 independent reflections
1955 reflections with *I* > 2σ(*I*)
*R*_int_ = 0.031

#### Refinement

*R*[*F* ^2^ > 2σ(*F* ^2^)] = 0.045
*wR*(*F* ^2^) = 0.142
*S* = 1.03
2802 reflections
201 parameters
H-atom parameters constrained
Δρ_max_ = 0.24 e Å^−3^
Δρ_min_ = −0.14 e Å^−3^

### 

Data collection: *APEX2* (Bruker, 2004 ▶); cell refinement: *APEX2* and *SAINT* (Bruker, 2004 ▶); data reduction: *SAINT* and *XPREP* (Bruker, 2004 ▶); program(s) used to solve structure: *SIR92* (Altomare *et al.*, 1994 ▶); program(s) used to refine structure: *SHELXL97* (Sheldrick, 2008 ▶); molecular graphics: *ORTEP-3* (Farrugia, 2012 ▶) and *CAMERON* (Watkin *et al.*, 1993 ▶); software used to prepare material for publication: *PARST* (Nardelli, 1995 ▶) and *WinGX* (Farrugia, 2012 ▶).

## Supplementary Material

Crystal structure: contains datablock(s) I, global. DOI: 10.1107/S1600536813000925/hg5275sup1.cif

Structure factors: contains datablock(s) I. DOI: 10.1107/S1600536813000925/hg5275Isup2.hkl

Supplementary material file. DOI: 10.1107/S1600536813000925/hg5275Isup3.cml

Additional supplementary materials:  crystallographic information; 3D view; checkCIF report

## Figures and Tables

**Table 1:** Hydrogen-bond geometry (Å, °) *Cg* is the centroid of the C11–C16 ring.

*D*—H⋯*A*	*D*—H	H⋯*A*	*D*⋯*A*	*D*—H⋯*A*
C18—H18*A*⋯*Cg* ^i^	0.96	2.87	3.755 (4)	154

Symmetry code: (i) 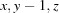.

**Fig. 1.**
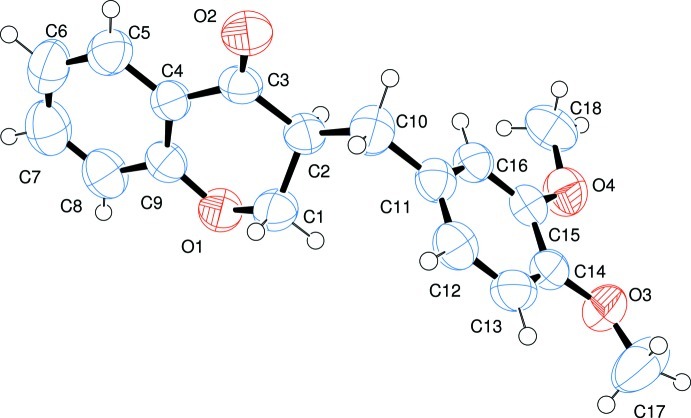
The molecular structure of the title compound, showing the atom-labeling scheme with displacement ellipsoids drawn at 50% probability level.

**Fig. 2.**
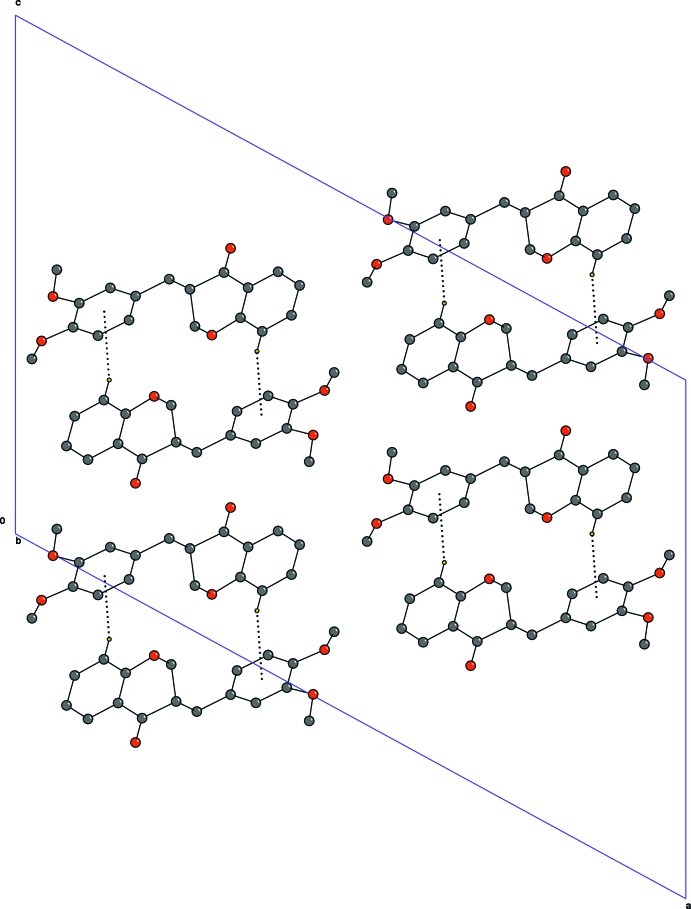
Packing diagram of (I) Dashed lines indicate C – H···π interactions. H-atoms not involving in H-bonding are omitted for clarity.

## supplementary crystallographic information

### Comment

Homoisoflavonones have wide spectrum of biological activity. In the past decades they are widely used as antifungal, antiviral, antimutagenic,antiproliferative, antioxidant, and protein tyrosine kinase (PTK) inhibitor activities (Jasinski *et al.*, 2010). Because of their wide range of pharmacological activity, the title compound (I) was synthesized and the crystal structure determined.

The bond distances and angles are found to have normal values (Allen *et al.*, 1987). The pyranone ring has adopted the envelope conformation (Cremer & Pople, 1975) as shown in Fig.1. The ring puckering parameters q_2_ = 0.3696 (2) Å,q_3_=0.2785 (3) Å, Q_T_ = 0.4628 Å, and φ = 276.96 (2)°, are indicative of an envelope conformation. The carbonyl ketone, being an electron-withdrawing group, makes the internal aromatic angle 118.17 (3)° at the C9 atom. The electronegative oxygen of the fused pyranone ring shows no change in the internal aromatic angle at the C5 atom. The six membered fused pyranone ring makes a dihedral angle of 89.72 (2)° with the dimethoxy substituted phenyl ring.

No classical inter- or intra-molecular hydrogen bonds are observed. The packing of the title compound is stabilized into a three-dimensional network by C—H···π intermolecular interactions, which serve to link inversion-related sheets (Fig 2).

### Experimental

2'-Hydroxydihydrochalcone (0.1 g) was dissolved in ethanol (10 ml) and refluxed with paraformaldehyde (0.022 g) and 50% aqueous diethylamine (0.2 ml) for 7 hrs. Ethanol was distilled off and the residue was taken up in ethyl acetate. The ethyl acetate layer washed with water then with dilute HCl and finally with water. Ethyl acetate was distilled off and the oily residue was column chromatographed over silica using pet ether(7): ethyl acetate(3) as eluent to get the 3-(3,4-dimethoxybenzyl)-2,3-dihydro-4*H*-chroman-4-one. Single crystals of the title compound were grown using methanol as solvent by slow evaporation technique and white needle-like crystals were harvested at room temperature. M.P. 397 K. Yield: 65%. IR(KBr):3001,2947,1689 cm^-1^. ^1^H-NMR,(400 MHz, DMSO) 4.3(dd,J=11.2,4.4Hz,1H,2-H), 4.2(dd,J=11.6, 9.2Hz,1H,2-H), 2.6(m,1H,3-H), 3.1(m,2H,9 -H), 3.7[S,6H,3 ,4 (2x OCH3)], 7.7(dd,J=8.1, 0.6Hz,1H, Ar-H), 7.5(m, 1H,Ar-H), 6.7(dd,J=8.4 ,2Hz,1H,Ar-H).

### Refinement

All H atoms were fixed geometrically and treated as riding with C—H = 0.95 Å (aromatic), 0.98 Å (methyl) or 0.99 Å (methylene) with *U*_iso_(H) = 1.2*U*_eq_(C) or *U*_iso_(H) = 1.5*U*_eq_(C_methyl_).

### Crystal data

**Table:**

C_18_H_18_O_4_	*F*(000) = 1264
*M**_r_* = 298.33	*D*_x_ = 1.317 Mg m^−^^3^
Monoclinic, *C*2/*c*	Mo *K*α radiation, λ = 0.71073 Å
Hall symbol: -C 2yc	Cell parameters from 575 reflections
*a* = 30.414 (4) Å	θ = 2.0–25.0°
*b* = 5.453 (3) Å	µ = 0.09 mm^−^^1^
*c* = 20.661 (5) Å	*T* = 295 K
β = 118.568 (3)°	Block, colourless
*V* = 3009.4 (19) Å^3^	0.30 × 0.20 × 0.20 mm
*Z* = 8	

### Data collection

**Table:**

Bruker Kappa APEXII CCD diffractometer	2802 independent reflections
Radiation source: fine-focus sealed tube	1955 reflections with *I* > 2σ(*I*)
Graphite monochromator	*R*_int_ = 0.031
ω and φ scan	θ_max_ = 25.5°, θ_min_ = 2.2°
Absorption correction: multi-scan (*SADABS*; Sheldrick, 1996)	*h* = −36→34
*T*_min_ = 0.954, *T*_max_ = 0.991	*k* = −6→6
13379 measured reflections	*l* = −13→25

### Refinement

**Table:**

Refinement on *F*^2^	Primary atom site location: structure-invariant direct methods
Least-squares matrix: full	Secondary atom site location: difference Fourier map
*R*[*F*^2^ > 2σ(*F*^2^)] = 0.045	Hydrogen site location: inferred from neighbouring sites
*wR*(*F*^2^) = 0.142	H-atom parameters constrained
*S* = 1.03	*w* = 1/[σ^2^(*F*_o_^2^) + (0.0617*P*)^2^ + 1.6626*P*] where *P* = (*F*_o_^2^ + 2*F*_c_^2^)/3
2802 reflections	(Δ/σ)_max_ < 0.001
201 parameters	Δρ_max_ = 0.24 e Å^−^^3^
0 restraints	Δρ_min_ = −0.14 e Å^−^^3^

### Special details

**Table:**

Geometry. All e.s.d.'s (except the e.s.d. in the dihedral angle between two l.s. planes) are estimated using the full covariance matrix. The cell e.s.d.'s are taken into account individually in the estimation of e.s.d.'s in distances, angles and torsion angles; correlations between e.s.d.'s in cell parameters are only used when they are defined by crystal symmetry. An approximate (isotropic) treatment of cell e.s.d.'s is used for estimating e.s.d.'s involving l.s. planes.
Refinement. Refinement of *F*^2^ against ALL reflections. The weighted *R*-factor *wR* and goodness of fit *S* are based on *F*^2^, conventional *R*-factors *R* are based on *F*, with *F* set to zero for negative *F*^2^. The threshold expression of *F*^2^ > σ(*F*^2^) is used only for calculating *R*-factors(gt) *etc*. and is not relevant to the choice of reflections for refinement. *R*-factors based on *F*^2^ are statistically about twice as large as those based on *F*, and *R*- factors based on ALL data will be even larger.

### Fractional atomic coordinates and isotropic or equivalent isotropic displacement parameters (Å^2^)

**Table:**

	*x*	*y*	*z*	*U*_iso_*/*U*_eq_	
O1	0.29295 (5)	−0.2335 (3)	0.08927 (7)	0.0703 (4)	
O2	0.32093 (6)	0.1349 (3)	0.27652 (7)	0.0795 (5)	
O3	0.03885 (6)	0.2688 (3)	−0.10089 (8)	0.0857 (5)	
O4	0.05593 (6)	−0.0630 (3)	−0.00297 (9)	0.0828 (5)	
C1	0.26773 (8)	−0.0139 (4)	0.08851 (10)	0.0695 (6)	
H1A	0.2353	−0.0109	0.0445	0.083*	
H1B	0.2869	0.125	0.0862	0.083*	
C2	0.26030 (7)	0.0125 (4)	0.15473 (10)	0.0591 (5)	
H2	0.2423	−0.1338	0.1569	0.071*	
C3	0.31104 (7)	0.0101 (4)	0.22267 (10)	0.0576 (5)	
C4	0.34695 (7)	−0.1611 (4)	0.21878 (10)	0.0558 (5)	
C5	0.39185 (8)	−0.2207 (4)	0.28105 (12)	0.0743 (6)	
H5	0.4002	−0.1446	0.3257	0.089*	
C6	0.42394 (9)	−0.3901 (5)	0.27737 (16)	0.0874 (8)	
H6	0.4537	−0.4293	0.3193	0.105*	
C7	0.41172 (10)	−0.5014 (5)	0.21116 (17)	0.0926 (8)	
H7	0.4336	−0.6151	0.2085	0.111*	
C8	0.36811 (9)	−0.4479 (4)	0.14941 (15)	0.0809 (7)	
H8	0.3602	−0.5257	0.1051	0.097*	
C9	0.33563 (8)	−0.2773 (4)	0.15279 (11)	0.0598 (5)	
C10	0.22947 (8)	0.2319 (4)	0.15249 (12)	0.0725 (6)	
H10A	0.2481	0.3796	0.1555	0.087*	
H10B	0.2245	0.2278	0.1955	0.087*	
C11	0.17901 (7)	0.2453 (4)	0.08429 (11)	0.0604 (5)	
C12	0.16944 (8)	0.4179 (4)	0.03117 (12)	0.0681 (6)	
H12	0.1945	0.5279	0.0371	0.082*	
C13	0.12308 (9)	0.4315 (4)	−0.03128 (12)	0.0692 (6)	
H13	0.1172	0.5511	−0.0666	0.083*	
C14	0.08591 (8)	0.2697 (4)	−0.04133 (11)	0.0626 (5)	
C15	0.09518 (7)	0.0896 (4)	0.01179 (11)	0.0602 (5)	
C16	0.14145 (7)	0.0798 (4)	0.07368 (11)	0.0608 (5)	
H16	0.1477	−0.04	0.1091	0.073*	
C17	0.02452 (12)	0.4796 (6)	−0.14567 (14)	0.1180 (11)	
H17A	0.0302	0.6225	−0.1155	0.177*	
H17B	−0.0103	0.4689	−0.1811	0.177*	
H17C	0.044	0.4912	−0.1709	0.177*	
C18	0.06175 (10)	−0.2336 (5)	0.05250 (14)	0.0907 (8)	
H18A	0.0881	−0.3467	0.0606	0.136*	
H18B	0.031	−0.322	0.037	0.136*	
H18C	0.0701	−0.1475	0.0974	0.136*	

### Atomic displacement parameters (Å^2^)

**Table:**

	*U*^11^	*U*^22^	*U*^33^	*U*^12^	*U*^13^	*U*^23^
O1	0.0715 (9)	0.0778 (10)	0.0596 (8)	0.0033 (8)	0.0297 (7)	−0.0169 (7)
O2	0.0778 (10)	0.0985 (12)	0.0547 (8)	−0.0048 (9)	0.0257 (7)	−0.0235 (8)
O3	0.0705 (10)	0.1063 (13)	0.0663 (9)	0.0163 (9)	0.0214 (8)	0.0078 (9)
O4	0.0645 (10)	0.0931 (12)	0.0908 (11)	−0.0091 (8)	0.0372 (8)	0.0017 (9)
C1	0.0716 (14)	0.0771 (14)	0.0552 (12)	0.0045 (11)	0.0266 (10)	−0.0073 (10)
C2	0.0586 (12)	0.0636 (12)	0.0540 (11)	−0.0042 (9)	0.0259 (9)	−0.0108 (9)
C3	0.0601 (12)	0.0652 (12)	0.0491 (10)	−0.0107 (9)	0.0275 (9)	−0.0071 (9)
C4	0.0520 (11)	0.0580 (11)	0.0575 (11)	−0.0085 (9)	0.0262 (9)	0.0008 (9)
C5	0.0647 (14)	0.0821 (15)	0.0700 (13)	−0.0081 (12)	0.0272 (11)	0.0052 (11)
C6	0.0614 (14)	0.0860 (17)	0.1025 (19)	0.0083 (13)	0.0293 (13)	0.0242 (15)
C7	0.0870 (18)	0.0789 (17)	0.120 (2)	0.0132 (14)	0.0564 (17)	0.0066 (16)
C8	0.0863 (17)	0.0707 (15)	0.0970 (17)	0.0048 (13)	0.0530 (15)	−0.0066 (13)
C9	0.0632 (12)	0.0564 (11)	0.0660 (12)	−0.0073 (9)	0.0359 (10)	−0.0035 (9)
C10	0.0687 (14)	0.0697 (14)	0.0707 (13)	0.0027 (11)	0.0264 (11)	−0.0177 (11)
C11	0.0624 (12)	0.0570 (12)	0.0642 (12)	0.0038 (10)	0.0321 (10)	−0.0080 (10)
C12	0.0704 (14)	0.0621 (13)	0.0795 (14)	−0.0028 (10)	0.0419 (12)	−0.0047 (11)
C13	0.0846 (16)	0.0658 (13)	0.0666 (13)	0.0117 (12)	0.0438 (12)	0.0099 (10)
C14	0.0615 (12)	0.0734 (14)	0.0552 (11)	0.0108 (11)	0.0297 (10)	−0.0025 (10)
C15	0.0589 (12)	0.0649 (12)	0.0665 (12)	0.0031 (10)	0.0378 (10)	−0.0032 (10)
C16	0.0662 (13)	0.0614 (12)	0.0594 (11)	0.0086 (10)	0.0338 (10)	0.0024 (9)
C17	0.128 (2)	0.112 (2)	0.0697 (16)	0.0409 (19)	0.0112 (15)	0.0049 (16)
C18	0.1060 (19)	0.0895 (18)	0.1038 (18)	−0.0211 (15)	0.0720 (16)	−0.0050 (15)

### Geometric parameters (Å, º)

**Table:**

O1—C9	1.355 (2)	C7—H7	0.93
O1—C1	1.418 (3)	C8—C9	1.383 (3)
O2—C3	1.213 (2)	C8—H8	0.93
O3—C14	1.371 (2)	C10—C11	1.509 (3)
O3—C17	1.408 (3)	C10—H10A	0.97
O4—C15	1.365 (2)	C10—H10B	0.97
O4—C18	1.420 (3)	C11—C12	1.368 (3)
C1—C2	1.496 (3)	C11—C16	1.388 (3)
C1—H1A	0.97	C12—C13	1.385 (3)
C1—H1B	0.97	C12—H12	0.93
C2—C10	1.507 (3)	C13—C14	1.369 (3)
C2—C3	1.510 (3)	C13—H13	0.93
C2—H2	0.98	C14—C15	1.397 (3)
C3—C4	1.468 (3)	C15—C16	1.377 (3)
C4—C9	1.389 (3)	C16—H16	0.93
C4—C5	1.395 (3)	C17—H17A	0.96
C5—C6	1.372 (3)	C17—H17B	0.96
C5—H5	0.93	C17—H17C	0.96
C6—C7	1.375 (4)	C18—H18A	0.96
C6—H6	0.93	C18—H18B	0.96
C7—C8	1.362 (3)	C18—H18C	0.96
			
C9—O1—C1	114.98 (15)	C2—C10—C11	114.02 (16)
C14—O3—C17	116.7 (2)	C2—C10—H10A	108.7
C15—O4—C18	117.46 (17)	C11—C10—H10A	108.7
O1—C1—C2	112.85 (17)	C2—C10—H10B	108.7
O1—C1—H1A	109	C11—C10—H10B	108.7
C2—C1—H1A	109	H10A—C10—H10B	107.6
O1—C1—H1B	109	C12—C11—C16	118.52 (19)
C2—C1—H1B	109	C12—C11—C10	120.9 (2)
H1A—C1—H1B	107.8	C16—C11—C10	120.57 (19)
C10—C2—C3	112.31 (16)	C11—C12—C13	121.0 (2)
C10—C2—C1	114.33 (18)	C11—C12—H12	119.5
C3—C2—C1	108.37 (16)	C13—C12—H12	119.5
C10—C2—H2	107.2	C14—C13—C12	120.4 (2)
C3—C2—H2	107.2	C14—C13—H13	119.8
C1—C2—H2	107.2	C12—C13—H13	119.8
O2—C3—C4	122.84 (18)	C13—C14—C15	119.46 (19)
O2—C3—C2	122.88 (19)	C13—C14—O3	124.8 (2)
C4—C3—C2	114.26 (16)	C15—C14—O3	115.75 (19)
C9—C4—C5	118.2 (2)	O4—C15—C16	125.46 (19)
C9—C4—C3	120.14 (17)	O4—C15—C14	115.26 (18)
C5—C4—C3	121.58 (18)	C16—C15—C14	119.28 (19)
C6—C5—C4	121.0 (2)	C15—C16—C11	121.32 (19)
C6—C5—H5	119.5	C15—C16—H16	119.3
C4—C5—H5	119.5	C11—C16—H16	119.3
C5—C6—C7	119.4 (2)	O3—C17—H17A	109.5
C5—C6—H6	120.3	O3—C17—H17B	109.5
C7—C6—H6	120.3	H17A—C17—H17B	109.5
C8—C7—C6	121.1 (2)	O3—C17—H17C	109.5
C8—C7—H7	119.5	H17A—C17—H17C	109.5
C6—C7—H7	119.5	H17B—C17—H17C	109.5
C7—C8—C9	119.7 (2)	O4—C18—H18A	109.5
C7—C8—H8	120.1	O4—C18—H18B	109.5
C9—C8—H8	120.1	H18A—C18—H18B	109.5
O1—C9—C8	116.51 (19)	O4—C18—H18C	109.5
O1—C9—C4	122.91 (18)	H18A—C18—H18C	109.5
C8—C9—C4	120.6 (2)	H18B—C18—H18C	109.5
			
C9—O1—C1—C2	−49.8 (2)	C3—C4—C9—C8	−176.84 (19)
O1—C1—C2—C10	−174.62 (16)	C3—C2—C10—C11	178.29 (17)
O1—C1—C2—C3	59.3 (2)	C1—C2—C10—C11	54.3 (3)
C10—C2—C3—O2	16.4 (3)	C2—C10—C11—C12	−108.8 (2)
C1—C2—C3—O2	143.6 (2)	C2—C10—C11—C16	70.3 (3)
C10—C2—C3—C4	−165.23 (17)	C16—C11—C12—C13	1.3 (3)
C1—C2—C3—C4	−38.0 (2)	C10—C11—C12—C13	−179.64 (19)
O2—C3—C4—C9	−172.37 (19)	C11—C12—C13—C14	−0.6 (3)
C2—C3—C4—C9	9.2 (3)	C12—C13—C14—C15	−0.4 (3)
O2—C3—C4—C5	10.7 (3)	C12—C13—C14—O3	179.69 (18)
C2—C3—C4—C5	−167.68 (18)	C17—O3—C14—C13	−14.9 (3)
C9—C4—C5—C6	−0.2 (3)	C17—O3—C14—C15	165.1 (2)
C3—C4—C5—C6	176.79 (19)	C18—O4—C15—C16	5.3 (3)
C4—C5—C6—C7	0.4 (4)	C18—O4—C15—C14	−174.00 (18)
C5—C6—C7—C8	−0.6 (4)	C13—C14—C15—O4	179.99 (18)
C6—C7—C8—C9	0.6 (4)	O3—C14—C15—O4	0.0 (3)
C1—O1—C9—C8	−162.74 (19)	C13—C14—C15—C16	0.7 (3)
C1—O1—C9—C4	17.7 (3)	O3—C14—C15—C16	−179.39 (17)
C7—C8—C9—O1	−179.9 (2)	O4—C15—C16—C11	−179.27 (18)
C7—C8—C9—C4	−0.4 (3)	C14—C15—C16—C11	0.0 (3)
C5—C4—C9—O1	179.68 (18)	C12—C11—C16—C15	−0.9 (3)
C3—C4—C9—O1	2.7 (3)	C10—C11—C16—C15	179.95 (18)
C5—C4—C9—C8	0.2 (3)		

### Hydrogen-bond geometry (Å, º)

Cg is the centroid of the C11–C16 ring.

**Table:**

*D*—H···*A*	*D*—H	H···*A*	*D*···*A*	*D*—H···*A*
C8—H8···*Cg*^i^	0.96	2.87	3.755 (4)	154

Symmetry code: (i) *x*, *y*−1, *z*.
